# Crystallisation of the amorphous ices in the intermediate pressure regime

**DOI:** 10.1038/s41598-017-03583-2

**Published:** 2017-06-21

**Authors:** J. Stern, T. Loerting

**Affiliations:** Institute of Physical Chemistry, University of Innsbruck, A-6020 Innsbruck, Austria

## Abstract

The crystallisation behaviour of very high-density amorphous ice (VHDA) and unannealed high-density amorphous ice (uHDA) has been studied *in situ* by volumetry and *ex situ* by powder x-ray diffraction in the intermediate pressure range 0.7–1.8 GPa employing different heating rates (0.5, 5 and 30 K min^−1^). This study shows that at pressures >1 GPa the crystallisation behaviour of VHDA and uHDA is basically the same for all heating rates. That is, parallel crystallisation is almost entirely suppressed with mainly ice XII forming. This contrasts former results reporting parallel crystallisation to approximately levelled phase mixtures of ice IV and ice XII even at higher pressures for uHDA. We speculate this to be due to formation of microcracks upon decompression in earlier works, but not in the present one. Crystallisation temperatures *T*_*x*_ are up to 16 K higher than previously reported, raising the low-temperature border to no man’s land and opening a considerably larger window for future studies on non-crystalline water. The results indicate uHDA to contain heterogeneities on the nanoscale, but VHDA to be rather homogeneous with nano-crystallites being largely absent. Upon transforming uHDA to VHDA, the nano-scale heterogeneities disappear for >1 GPa whereas microcracks do not.

## Introduction

**Figure 1:**
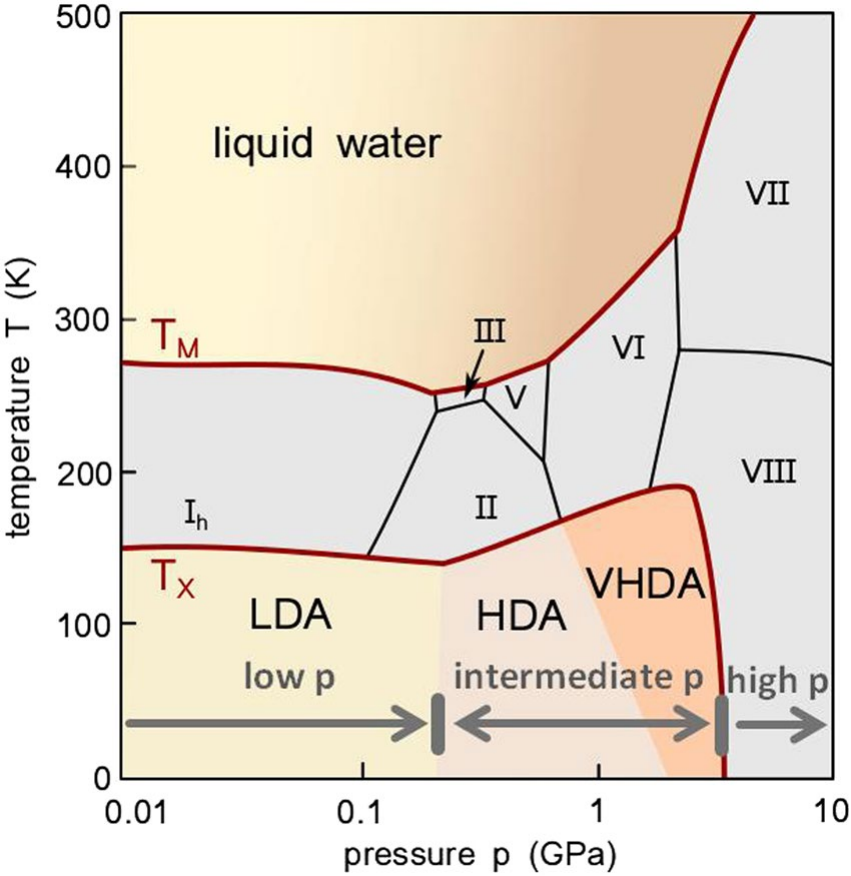
Phase diagram of water including the range of observation for metastable amorphous ices. The area of stability for the crystalline phases is bordered by the melting line T_M_ at higher temperatures and the crystallisation line of the amorphous solid phases T_X_ at low temperatures. LDA is mostly found in the low-pressure region of the phase diagram (0–0.2 GPa), HDA and VHDA in the intermediate-pressure region (~0.2–2 GPa). At high pressures (>2 GPa) only crystalline phases are stable. Adapted from^43^.

Water is a fascinating substance and in many ways its behaviour eludes scientific expectations. These aberrations from the ‘norm’ have been labelled the anomalies of water and have been described many times in literature^1–3^. One of its puzzling qualities is the great variety of solid phases it can form. Besides the numerous solid crystalline types (polymorphism)^4, 5^ also different solid amorphous phases have been identified (polyamorphism)^6, 7^. Figure 1 shows the phase-diagram of the thermodynamically stable phases and additionally includes the pressure-temperature regions, in which amorphous ices have been identified. By contrast to the other phases shown in the phase-diagram the amorphous ices are metastable, i.e., there is a phase of crystalline ice lower in Gibbs free energy. In this phase diagram three pressure regimes can be broadly defined. The lower pressure regime can be related to the area of stability of hexagonal ice I_h_ and at lower temperatures of ice XI, i.e., ~0–0.2 GPa. Intermediate pressures^8^ (also referred to as “medium pressures” in the literature^9^) range from ~0.2–2 GPa. This is the richest pressure range, in which water exhibits a broad variety of stable and metastable crystalline phases (ices II–VI, ice IX and ices XII–XV). The higher pressure range is accordingly located at ~*p* > 2 GPa where the “symmetric” ices VII, VIII and X can be prepared^8^. The amorphous ices have been labelled according to their densities and may occur below *T* ≈ 180 K and *p* < 3.5 GPa. Amorphous ice of low density can be obtained by water vapour deposition on a cold substrate^10^, hyperquenching of micrometer-sized droplets onto a cryoplate^11^ or by decompression of high-density amorphous ice (HDA) at elevated temperatures^12^. The low-density amorphous ices (LDAs) prepared by these different routes are very similar in terms of structure and density, and thus they represent a thermodynamically well defined metastable phase^13^. HDA was discovered in 1984 by Mishima *et al*.^12^ by pressurisation of hexagonal ice I_h_ at liquid nitrogen temperatures. This process of pressure induced amorphisation (PIA) was then observed for the first time on the example of water. The latest amorphous phase described is very high-density amorphous ice (VHDA^14^). It can be obtained by annealing of HDA at intermediate pressures (*p* > 0.8 GPa). Just like all stable and metastable crystalline ices, the amorphous ices can be quenched and recovered at ambient pressure and stored in liquid nitrogen. At atmospheric pressure and 77 K the amorphous ices’ densities are 0.94 ± 0.02 g cm^−3^ (LDA^15^), 1.17 ± 0.02 g cm^−3^ (HDA^12, 15^) and 1.25 ± 0.01 g cm^−3^ (VHDA^14^). LDA is located in the lower pressure regime, while HDA and VHDA are situated in the intermediate pressure regime and cease to exist at pressures exceeding ~3 GPa^16^. Just like between crystalline ices it is possible to switch back and forth between amorphous ices by varying pressure. One has to note, though, that the pressure-induced interconversion of LDA into HDA and HDA into LDA is accompanied by a hysteresis, i.e., there is a range of metastability in the low-pressure regime for HDA and likewise in the intermediate-pressure regime for LDA^17, 18^.

The existence of more than one amorphous solid form of water is directly connected to the hypothesis that more than one liquid phase may exist for water. This hypothesis was described first in simulation studies and included the possible existence of a second critical point^19^. It aims to explain the abnormal evolution of various thermodynamic quantities in a region of the phase diagram which has been rather elusive to experiments. This region has been labelled ‘no man’s land’ of water^20^, a term originally “used to define a contested territory”^21^ and especially connected to the unoccupied fighting ground between the opposing forces in World War I. Regarding water, no man’s land is bordered by the homogeneous nucleation at higher temperatures T_H_ where liquid water freezes inevitably and at lower temperatures by the crystallisation temperature of the amorphous ice phases T_X_ (Fig. 1). Thus it is actually not an empty land in the original sense of the latin “terra nullius”, but occupied by ice polymorphs as is illustrated in Fig. 1. Only when considering water’s non-crystalline phase diagram (i.e., by excluding ice polymorphs) it can be referred to as unoccupied. However, even in this sense the term ‘no man’s land’ is used differently from its original usage: instead of two antagonising parties bordering to the ‘no man’s land’, rather one party, namely supercooled water, is bordering to it from both sides. It is in this region where the location of a possible second critical point and a phase boundary between two distinct liquid forms of water has been postulated^19^. One has to note, though, that there is still an ongoing discussion regarding the relation of the amorphous ices between one another and their relation to supercooled liquids^22^.

Different routes have been taken to breach or overcome the boundaries to no man’s land, both in experiments as well as in simulations^23, 24^. Coming from the high-temperature side crystallisation of liquid water has been delayed or avoided by hyperquenching experiments^11^, studying it in confinement^25, 26^ or as nanodroplets^27^. It may be disputable however, how far nanoscopic volumes interacting with the confining surfaces actually resemble bulk water. In bulk droplets fast evaporative cooling was coupled with ultrafast laser probing in order to obtain information about structure^28^ and nucleation rates near T_H_ down to 227 K^29^. Near T_X_ isobaric heating experiments were done on LDA by differential thermal analysis (DTA)^17^ and on HDA by *in situ* volumetry^30–32^. In the study performed by Seidl *et al*. mainly in the low-pressure regime^31, 32^ it was shown that the pressure annealed variant of high-density amorphous ice – expanded HDA (eHDA) – is up to 11 K more stable against crystallisation than the HDA obtained from pressure-induced amorphisation of ice I_h_ (unannealed HDA, uHDA). It was reasoned that the lower thermal stability of uHDA is due to nano-crystalline domains that remain in the amorphous matrix when it is produced from ice I_h_^31^. When uHDA is annealed at elevated pressures >1 GPa and decompressed at elevated temperatures to form eHDA the crystalline domains might disappear. In this scenario crystalline seeds are already prevalent in uHDA and only have to grow upon heating. In eHDA however, they first have to nucleate and then to grow, resulting in a higher thermal stability against crystallisation.

In the present study the work of Seidl *et al*. was extended to higher pressures by investigating crystallisation of uHDA and VHDA in the intermediate pressure range. The motivation is to study whether or not the pressure-annealing - that likely results in the disappearance of nano-crystalline domains responsible for the shrinking of the no-man’s land at low pressures - also helps to shrink the no-man’s land at intermediate pressures. Alternatively, the structural inhomogeneities related to nano-crystalline domains in uHDA may survive the process of annealing and still be prevalent in VHDA. As eHDA is prepared from VHDA when decompressing at elevated temperatures the nanocrystallites may very well disappear during the transformation of VHDA to eHDA. Similar to the experiments on uHDA and eHDA^30–32^ the crystallisation behaviour of VHDA was investigated *in situ* by volumetry and *ex situ* by powder x-ray diffraction. As we will demonstrate below, in the range *p* = 0.7–1.8 GPa VHDA behaves rather differently from uHDA as indicated in results ﻿previously ﻿reported﻿﻿in literature^30^. Notably, the process of parallel crystallisation appears to be much more suppressed in VHDA and crystallisation temperatures *T*_*x*_ seem to be considerably higher. By conducting an own set of experiments on uHDA under comparable conditions it can be shown however, that uHDA and VHDA exhibit almost the same crystallisation behaviour at *p* > 1 GPa. That is, uHDA reaches a state very similar to that of VHDA prior to transformation. At pressures *p* ≤ 0.8 GPa though, the results provide evidence for the suggestion of there being nano-crystalline ‘seeds’ in the amorphous matrix of uHDA. Effectively, due to these novel results water’s low-temperature boundary to the no-man’s land is raised by up to 16 K as compared to values formerly presented in literature^30^.

## Experimental and Analysis

For the sample preparation a custom made piston cylinder setup (8 mm bore diameter) was employed. Uniaxial pressure was applied using a computerised commercial material testing machine (ZWICK model BZ100/TL3S) which also allows the tracking of the piston displacement, and hence sample volume, *in situ* (software TESTXPERT 7.1). Displacement curves were converted into curves of volume change (Δ*V*) by taking into account the bore geometry. Temperature was controlled by heating elements inserted into separate bores in the cylinder close to the sample. Additionally, a stream of liquid/gaseous nitrogen pumped through copper loops surrounding the steel cell was regulated accordingly. The temperature was recorded *via* a temperature sensor (Pt-100) inserted into the steel cylinder in another separate bore close to the sample^33^.

**Figure 2:**
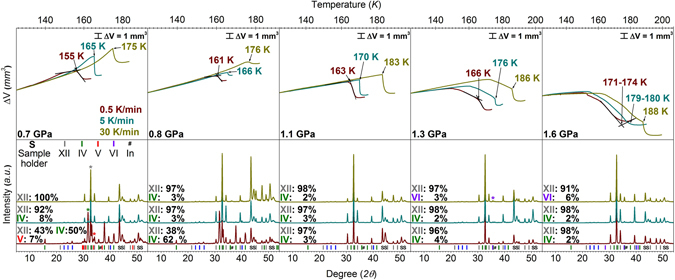
Isobaric heating of VHDA at different pressures *p* = 0.7–1.6 GPa. Samples were heated with different rates (depicted in respective colours) of 0.5 (dark red), 5 (cyan) and 30 K min^−1^ (dark yellow). In the upper panel the volume curves *ΔV* vs. temperature *T* are shown. Crystallisation temperatures *T*_*x*_ were usually identified by rather steep steps in the volume curve indicating transformation to denser crystalline phase mixtures and are labelled with the respective temperature values. In the lower panel powder x-ray diffractograms recorded *ex situ* are shown. The results from separate experiments with different heating rates are stacked for clarification. Relative yields of crystalline ices in the phase mixtures are indicated by percentages and the respective most intensive﻿ *Bragg* peaksmarked in the diffractograms with different colours (ice V in red, ice IV in green, ice XII in grey and ice VI in purple).

Crystallisation temperatures of very high-density and unannealed high-density amorphous ice were determined by conducting isobaric heating experiments. The starting material VHDA was prepared *via* uHDA, which itself was obtained by pressure-induced amorphisation of hexagonal ice I_h_. In detail, ice I_h_ was compressed to 1.6 GPa at 77 K to yield uHDA, then decompressed to 1.1 GPa and subsequently heated isobarically at 1.1 GPa to 160 K. Then the so-produced VHDA was quenched to 77 K and finally brought to selected pressures between 0.7 and 1.8 GPa. At these low temperatures VHDA does not reconvert to HDA even when going to pressures *p* ≤ 0.8 GPa^34^. VHDA (Fig. 2) was heated isobarically past the crystallisation temperature *T*_*x*_, were the phase transformation to crystalline forms (ices IV, V, VI and XII, respectively) was observed. Due to the difference in density between VHDA and the obtained crystalline phases (*ρ* (VHDA) < *ρ* (cryst. ice)), a given transformation could be identified by a rather steep ‘step’ in the displacement curve indicating sudden densification. Onset temperatures of crystallisation *T*_*x*_ were determined by the intersection of two straight lines (as indicated in Fig. 2 by the black tangents on the dark red volume curves when heating with 0.5 K min^−1^).

The samples were then quenched and extracted at ambient pressure for further structural analysis. Powder X-ray diffractograms were recorded in a vacuum chamber at ~80 K in *θ*-*θ* geometry (Siemens diffractometer, model D5000, Cu *Kα*). For quantitative analysis of the powder x-ray diffractograms the most intensive *Bragg* peaks (100% relative intensity, Table 1) of a given crystalline phase pattern were evaluated assuming sphere-like crystalline particles of small size. By comparing the most intensive *Bragg* peaks of different crystalline ices from one measurement phase compositions were determined.

**Table 1:** Most intensive peaks (100% relative intensity) of the respective crystalline ice phases. Structural data were taken from literature^39–42^.

Crystalline phases	IV	V	VI	XII
Most intense *Bragg* peak, 100% relative intensity (degree *2θ*)	31.6	33.8	35.5	32.8
Literature	39	40	41	42

## Results and Discussion

### Influence of heating rate and pressure

Similar to results formerly obtained on uHDA (0.2–1.9 GPa^30^; 0.1–0.5 GPa^32^) and eHDA (0.1–0.5 GPa^32^) VHDA experiences parallel crystallisation at intermediate pressures 0.7–1.8 GPa. That is, more than one polymorph of crystalline ice is formed upon crystallising VHDA. Figure 2 illustrates the results obtained in the pressure range 0.7–1.6 GPa for isobaric crystallisation of VHDA using three different heating rates. It is apparent that the crystalline phase compositions depend on pressure and heating rate. At *p* < 1.6 GPa VHDA mainly transforms to ices IV and XII, with some ice V forming at 0.7 GPa (0.5 K min^−1^). At pressures *p* ≥ 1.3 GPa ice VI is also found upon crystallisation (Fig. 2, lower part). Also, the crystallisation temperature *T*_*x*_ increases with increasing heating rate. At 1.1 GPa for instance, when heating with a low rate (0.5 K min^−1^) crystallisation occurs at a temperature 18.5 K lower (164.5 K) as compared to heating with a high rate (30 K min^−1^, 183 K).

At one given heating rate *T*_*x*_ also increases with increasing pressure. For a rate of 0.5 K min^−1^ and going from 0.7 → 1.6 GPa *T*_*x*_ is higher by approximately 15 K. The same holds true for intermediate (5 K min^−1^) and fast heating (30 K min^−1^). Furthermore, analogous to the former results on eHDA^31, 32^ and uHDA^30–32^, different mixtures of crystalline polymorphs are obtained. Especially at lower pressures (0.7 and 0.8 GPa) there is a considerable difference in the relative yields of crystalline phases when changing from slow to intermediate and fast heating. At 0.7 GPa ice IV (50%) and ice XII (43%) are formed in roughly equal amounts after heating with 0.5 K min^−1^. However, at 5 K min^−1^ mostly ice XII is obtained (92%) and far less ice IV (8%). At 30 K min^−1^ formation of ice IV is entirely suppressed, and ice XII can be obtained purely. While the observation of parallel crystallisation kinetics at elevated pressures is in qualitative agreement with previous results^30–32^, the suppression of all competing crystallisation channels even at low heating rates in the amorphous matrix and especially at pressures *p* ≥ 1.1 GPa is a novel result. Parallel crystallisation from the amorphous matrix is generally governed by processes with different kinetics: slower processes starting at lower temperatures and faster ones commencing at higher temperatures (labelled type 1 and 2^30^). Accordingly, at 0.7 GPa formation of ice IV (and V at a rate of 0.5 K min^−1^) on the one hand is governed by a process with slower kinetics, thus starting at lower temperatures and predominantly when heating with lower rates. On the other hand, formation of ice XII is determined by a process with faster kinetics. It crystallises to a greater extent at higher temperatures and faster heating rates. Similar behaviour is observed at 0.8 GPa where considerable amounts of ice IV crystallise only when heating with a low rate (62%, 0.5 K min^−1^; 38% ice XII)., while ice V does not form at all. At higher rates (5 and 30 K min^−1^) ice XII forms almost exclusively with a relative yield of >90%. The suppression of parallel crystallisation processes in VHDA is also evident at pressures above 0.8 GPa. Ice XII forms predominantly at 1.1, 1.3 and 1.6 GPa, no matter the heating rate. Its relative yield is >95% in nearly every case. Furthermore, at *p* ≥ 1.3 GPa also ice VI is obtained at a high heating rate. At 1.8 GPa (not included in Fig. 2, as only one heating rate was studied at this pressure) when heating with 5 K min^−1^ ice VI takes over as the predominant phase forming upon crystallisation (83% ice VI, 17% ice XII).

This fits the picture established in the work reported on uHDA^30^. That is, the process of parallel crystallisation can be characterised by two (or more) different kinetics associated with polymorphic formation. At lower heating rates crystallisation occurs at lower temperatures and phases that form with slower kinetics will be obtained to a greater extent. These slower kinetic processes govern the formation of ices IV and V at lower pressures (Fig. 2). Ice V shows the slowest kinetics, and it only appears in traces at the lowest heating rate and lowest pressure studied. Correspondingly the transformation to ice XII is related to a process with faster kinetics. Thus ice XII is obtained at higher heating rates where *T*_*x*_ is also shifted to higher temperatures. This holds true for pressures up to 1.3 GPa. From there on ice VI starts to form when heating with 30 K min^−1^. The relative yield of ice VI increases with increasing pressure and at 1.8 GPa it is the phase of highest relative yield. That is, at lower pressures *p* ≥ 0.7 GPa the transformation to ice XII is the process with the fastest kinetics. At higher pressures *p* ≥ 1.3 GPa formation of ice VI takes over as the process with fastest kinetics.

### Comparing *T*_*x*_ for VHDA and uHDA

**Figure 3:**
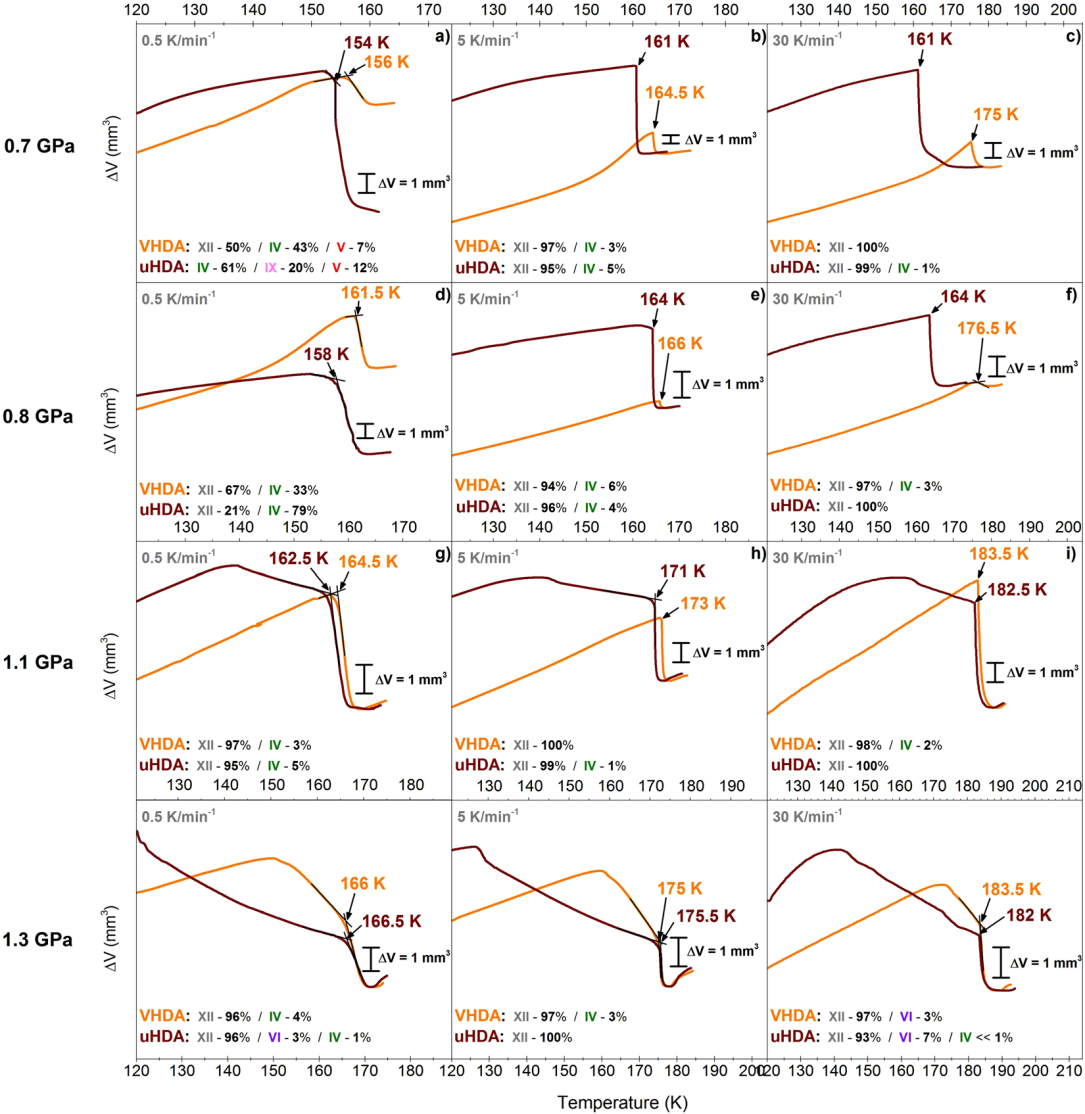
Volume curves of isobaric heating experiments with different heating rates (0.5, 5 and 30 K min^−1^) for uHDA (brown) and VHDA (orange) at pressures between 0.7 and 1.3 GPa. Crystallisation temperatures *T*_*x*_ were identified by rather steep steps in the volume curve indicating transformation to denser crystalline phase mixtures and are labelled with the respective temperature values. Relative yields of crystalline ices in the phase mixtures are indicated by percentages and the various polymorphs coded in different colours (ice IX in pink, ice V in red, ice IV in green and ice XII in grey).

While this behaviour is generally in accordance with the findings for uHDA^30^, there are quantitative differences regarding phase composition after transformation and especially the crystallisation temperature *T*_*x*_. At 0.71 GPa when heating with a rate of 0.5 K min^−1^ uHDA was reported to transform to ice IX before crystallising to ices IV and V (Fig. 3 in the work of Salzmann *et al*.^30^). Two density steps are observable in the Δ*V* curve, one attributable to the formation of ice IX and the other to the formation of ices IV and V. In VHDA ices IV, V and XII form simultaneously. To elucidate these differences between the amorphous ices, uHDA was investigated in a likewise manner in a pressure range from 0.7 to 1.3 GPa employing heating rates of 0.5, 5 and 30 K min^−1^. The results demonstrate that notable discrepancies between uHDA and VHDA as starting materials in fact only occur at pressures p < 1 GPa. In Fig. 3 the volume curves for uHDA and VHDA are presented for all pressures and heating rates. In the most instances the phase predominantly produced is ice XII, and it can generally be stated that the higher the pressure the more readily ice XII is formed. At 0.7 and 0.8 GPa crystallisation temperatures are in fact very similar for uHDA and VHDA at low and intermediate heating rates (0.5 and 5 K min^−1^), with a maximum difference of about 3.5 K at 0.7 GPa and heating with 5 K min^−1^. However, when high heating rates of 30 K min^−1^ are employed *T*_*x*_ is considerably lower for uHDA compared to VHDA, with a difference Δ*T*_*x*_ of 12.5 K at 0.8 GPa and even 14 K at 0.7 GPa (Fig. 3(c and f)). The general trend that *T*_*x*_ at a given pressure increases with the heating rate is not followed in the case of uHDA at 0.7 GPa and 0.8 GPa. Also, at 0.7 GPa and a low heating rate ice IX is formed, while ice XII is not formed at all. In contrast, for VHDA ice XII can be found on the one side in approximately equal amounts as ice IV (Fig. 3(a)), and on the other side ice IX is not produced. Seidl *et al*.^32^ have reasoned that nano-crystalline hexagonal seeds may indeed transform to ice IX when uHDA is heated isobarically at *p* ≥ 0.3 GPa. This may explain why ice IX forms even at pressures up to 0.7 GPa from uHDA, while it does not crystallise from VHDA at all. Furthermore, this may also be the reason for the substantial discrepancies in *T*_*x*_ at 0.7 and 0.8 GPa when heating with 30 K min^−1^. While it is possible that at low and intermediate heating rates the uHDA matrix and the nano-crystalline domains within have enough time to relax and transform to a state similar to that of VHDA during annealing, this may not be the case at a high heating rate.

**Figure 4:**
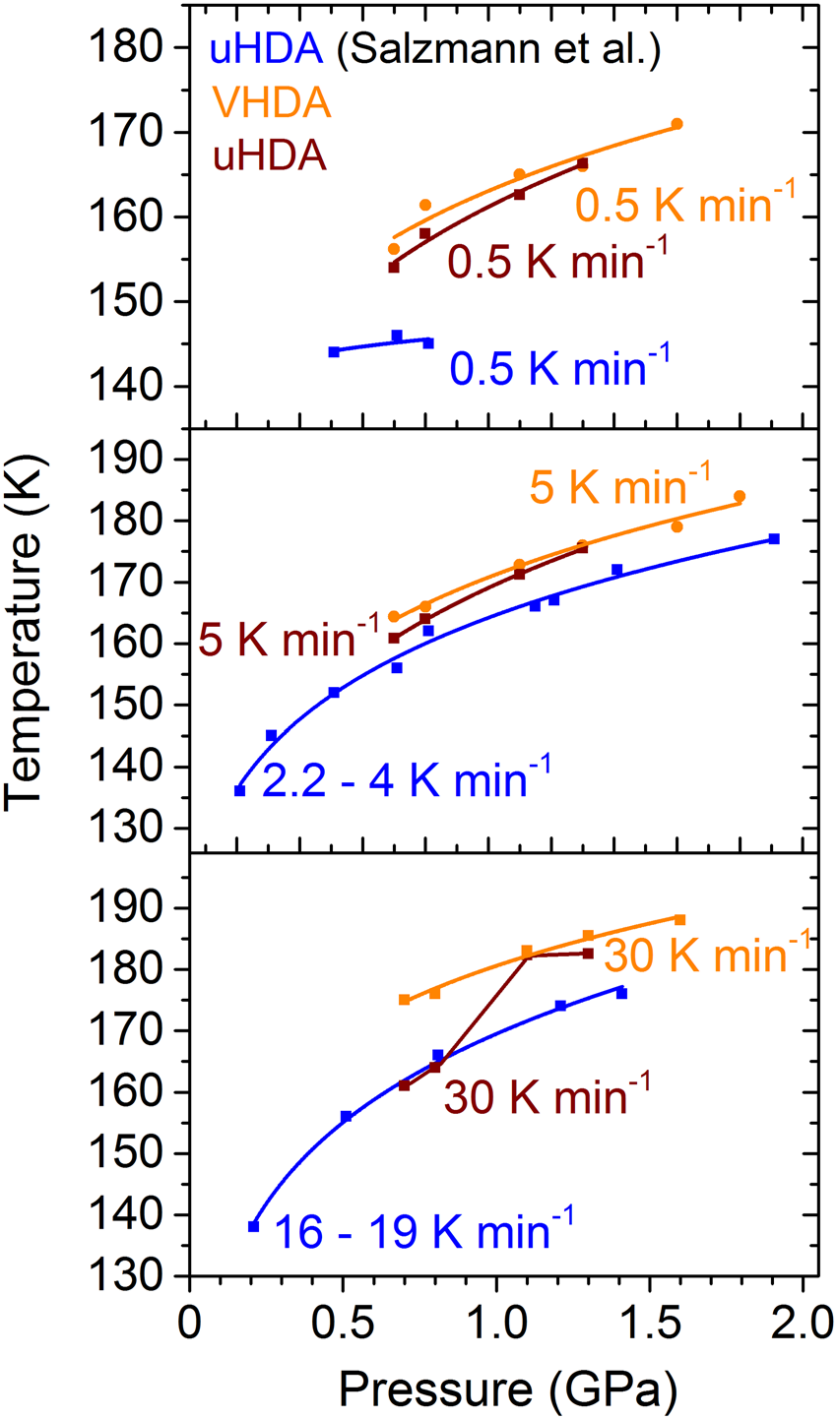
Crystallisation temperatures *T*_*x*_ of VHDA (orange) are compared to those of uHDA (brown) and to literature^30^ (blue) for (**a**)) slow, (**b**)) intermediate and (**c**)) fast heating.

**Figure 5:**
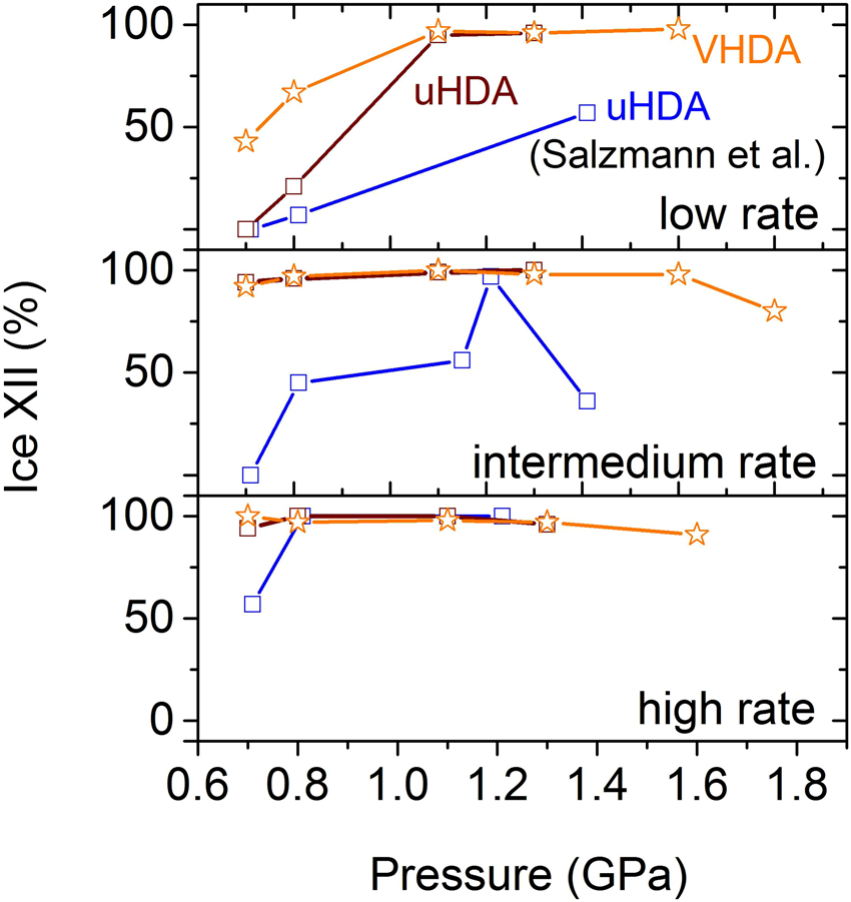
Relative yield of ice XII in crystalline phase mixtures at given pressures and heating rates. Values obtained after crystallisation from VHDA are coloured in orange, from uHDA in brown and values from literature in blue.

At pressures p > 1 GPa the differences in crystallisation behaviour disappear almost entirely. In fact, it appears that the higher the pressure, the smaller the difference between uHDA and VHDA both regarding *T*_*x*_ and the crystalline phase composition. At all pressures and heating rates ice XII is formed almost exclusively (>90%). This indicates that the structural states of the amorphous phases at one given pressure and when heating with one given rate is very similar, if not the same just before the transformation. A further evidence for this is that the densification steps upon transformation in the volume curves are of almost the same size, meaning that the density of the amorphous phases just prior to crystallisation is in fact very similar. This seems sensible, as one would expect uHDA to transform to VHDA at pressures p > 0.8 GPa already at temperatures *T* < *T*_*x*_. The uHDA to VHDA transition has been shown to take place at 130 K and 1.1 GPa by Amann-Winkel (Ph.D. thesis, Figs 4 and 5), i.e., above 130 K uHDA no longer exists. Though, what is not so clear is whether the nano-crystalline domains in uHDA suggested by Seidl *et al*. survive the uHDA to VHDA transition or not. The results presented above suggest that nano-crystalline domains indeed disappear in all cases for *p* > 1 GPa, and also for lower heating rates at lower pressures.

### Crystallisation behaviour of uHDA in comparison with literature

Overall, one can state that the differences in crystallisation behaviour between uHDA and eHDA noted at lower pressures p ≤ 0.5 GPa^31, 32^ can barely be noted when comparing uHDA to VHDA at higher pressures *p* ≥ 0.7 GPa. That is, only at pressures *p* < 1 GPa there are notable differences in phase composition (0.7 and 0.8 GPa when heating with 0.5 K min^−1^, see Fig. 3(a and d)) and crystallisation temperature (0.7 and 0.8 GPa when heating with 30 K min^−1^, see Fig. 3(c and f)).

When comparing the results obtained here on uHDA with those from literature^30^, however, the difference is quite substantial. These discrepancies are illustrated in Figures 4 and 5. As can be seen in Fig. 4 crystallisation temperatures for uHDA in literature are almost always below those found for uHDA in this study. This may partially be due to the fact that slightly different heating rates were employed in the literature. Nevertheless, this cannot account for the rather substantial variation especially at low heating rates 0.5 K min^−1^ where the heating rate was exactly the same.

Inspecting the literature data on uHDA crystallisation more carefully, two differences to the present study surface: (i) different definitions for *T*_*x*_ were utilised to mark the onset temperature of transformation on volume curves and (ii) a different protocol of preparation was used to prepare uHDA, which was first decompressed to ambient pressure and then recompressed before isobaric heating rather than directly decompressed as done in this study.

Concerning item (i), evaluating the old data with the tangent method also used in the present study the differences in *T*_*x*_ for instance at intermediate heating rates (2.2–4 and 5 K min^−1^, respectively) become almost negligible. However, at low heating rates of 0.5 K min^−1^ the variation in crystallisation temperature is still considerable even when taking into account different evaluation methods. One has to bear in mind that uHDA in literature^30^ and in this study were heated isobarically under nearly identical conditions (with 0.5 K min^−1^ at 0.71 and 0.81 GPa in literature instead of 0.7 and 0.8 GPa, see Fig. 4(a)).

Also the examination of the crystalline phase composition reveals large differences, even though the same starting material uHDA is compared, and the pressure and heating rate are practically identical. To illustrate these divergences relative yields of ice XII in the crystalline phase mixture are presented in dependence of the pressure in Fig. 5. While VHDA and uHDA in the underlying study crystallise to almost exclusively ice XII at all pressures and heating rates (notable exceptions being 0.7 and 0.8 GPa when heating with a low rate), the formation of ice XII in the literature^30^ does not follow a stringent pattern. Even at pressures *p* > 1 GPa the relative yield of ice XII may be approximately 50% or less (Fig. 5(b)).

That is, the different evaluation method in item (i) cannot explain these variances. Hence, the difference has to be sought for in the experimental protocol, i.e., in item (ii) mentioned above. While in this study uHDA was prepared from ice I_h_ and then brought to the respective pressure of isobaric heating immediately, the uHDA from literature was first decompressed to ambient pressure and subsequently recompressed to higher pressures. It has been shown that upon decompressing high-pressure ices to ambient pressure ‘(micro)cracks’^34, 35^ are introduced. These microcracks increase the surface area and may act as nucleation spots in the amorphous matrix and could very well be a cause for inhomogeneous crystallisation behaviour. In other words, the ice IV that was found to crystallize almost purely in literature in the slow heating experiments starts to crystallize from the additional surfaces within the microcracks. By contrast, ice IV cannot crystallize in the present work in similarly slow heating experiments because the microcracks and hence the nucleation sites for ice IV are absent.

### Relaxation of amorphous matrix vs. crystallisation in VHDA

**Figure 6:**
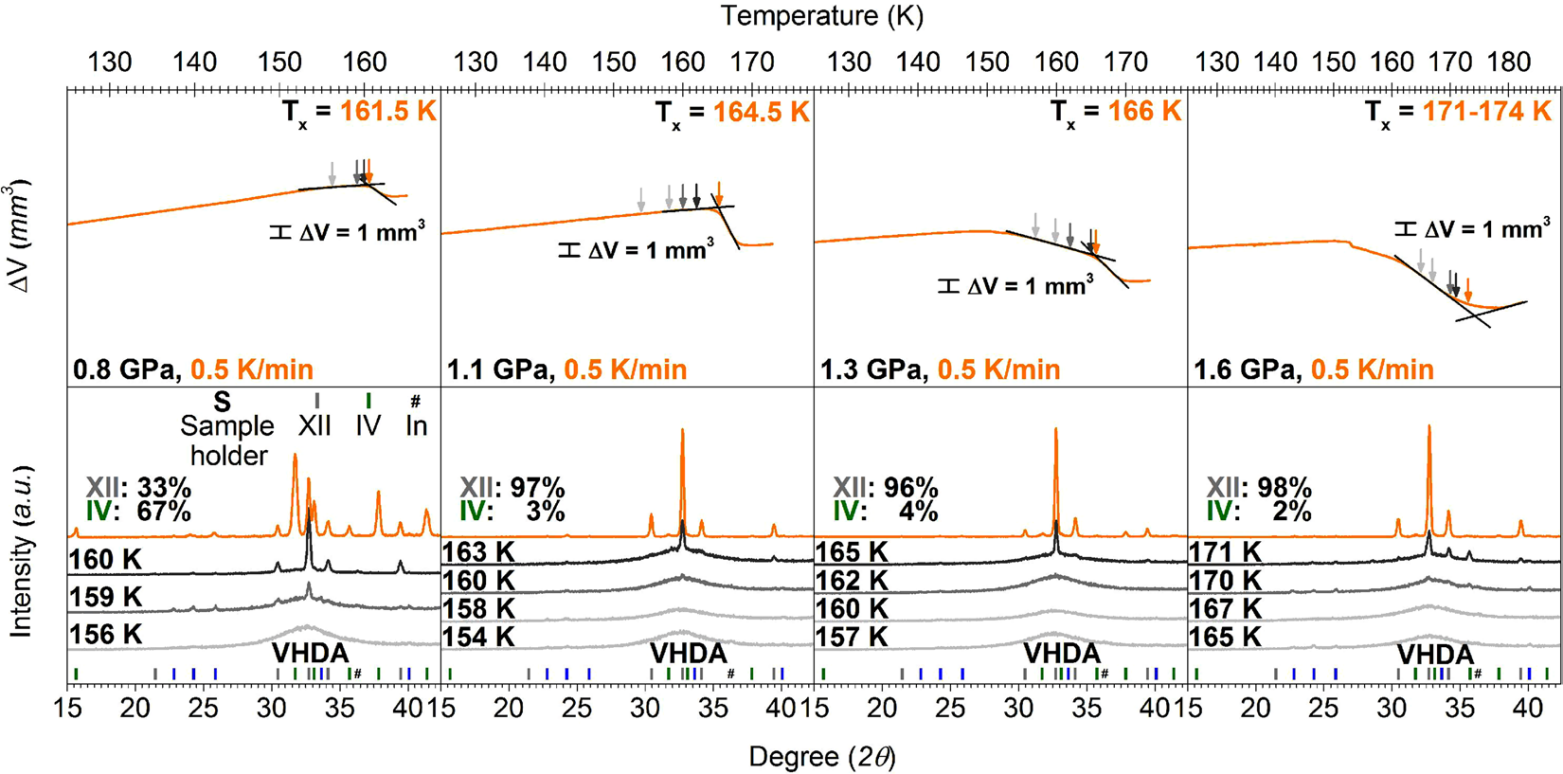
*Ex situ* x-ray characterization (bottom panels) of samples obtained at various points in volumetric experiments (top panels). For the volumetric experiments samples of VHDA were heated isobarically in a series of experiments at each pressure with 0.5 K min^−1^. Arrows in different grey scales identify temperatures at which samples were quenched and recovered for x-ray characterization. The orange arrow marks *T*_*x*_. Diffractograms are stacked for clarity and their colours match the colours of the arrows. The relative yields of crystalline ices in the phase mixtures are indicated by percentages and marked in different colours (ice IV in green and ice XII in grey).

*T*_*x*_ can be identified with ease for experiments at 1.1 GPa: the volumetric curves shown in Fig. 2 feature two straight lines and a sharp kink signalling *T*_*x*_. The two straight lines indicate linear thermal expansion of VHDA and sudden densification caused by crystallisation. This is evidenced in Fig. 6, for which the volumetric curves﻿ for a series of experiments at each pressure was analysed by *ex situ* X-ray diffraction experiments marked by arrows. Clearly, Bragg peaks suddenly appear at the kink, but not before the kink. At 0.7 and 0.8 GPa *T*_*x*_ can also be identified readily. The linear thermal expansion at low temperatures is followed by a stronger expansion at higher temperatures, where the amorphous matrix relaxes structurally and volumetrically in the experimental time scale. The sudden change from expansion to densification signals *T*_*x*_. After the complete transformation to a crystalline phase/phase mixture the sample thermally expands upon further increase of temperature. Determination of *T*_*x*_ is less straightforward at 1.3 and 1.6 GPa. The reason being that relaxation of the amorphous matrix is associated with densification (rather than the expansion seen at 0.7 GPa and 0.8 GPa). That is, densification may signal either relaxation of VHDA itself or crystallisation of VHDA. In most cases, the time scale for relaxation is much longer than the time scale for crystallisation. As a result one can identify two kinks in the volumetric experiment (see Δ*V* curves in Fig. 2), where the first one is associated with onset of VHDA relaxation and the second one with *T*_*x*_. However, especially at 1.6 GPa it is rather challenging to identify values for *T*_*x*_, most likely because the time scales for relaxation at the high temperature part of the curve become similar to the time scales for crystallisation. The result is a volumetric curve without a clear kink. The *ex situ* X-ray diffraction patterns (Fig. 6, 0.5 K min^−1^) show that transformation takes place just before the slope of the Δ*V* curve becomes positive again. That is, the expected step in the volume curve indicating crystallisation is masked by the densification of VHDA due to thermal relaxation. It is evident from the broad halo peak in the diffractograms that even at 170 K the sample is mainly amorphous. Only at 171 K sharp Bragg reflexes indicate the start of crystalline ice XII growth from the amorphous matrix. The change of slope in the Δ*V* curve from negative to positive reflects the completion of thermal relaxation and subsequent crystallisation of VHDA. The temperature, at which crystallisation commences can be extracted in this curve by the method of tangent intersection (see Fig. 6), which also results in 171 K. In this case there is a strong rounding of the volumetric curve near the tangent intersection, which shows that relaxation and crystallisation time scales are similar in this example. In most other cases the tangents coincide with the volume curves and signal that the time scales for relaxation and crystallisation are well separated, i.e., a well-defined kink is observed.

## Summary and Outlook

We have demonstrated in this study of isobaric heating experiments that VHDA exhibits a considerably different crystallisation behaviour from that previously reported for uHDA in literature^30^ in the intermediate pressure range. Especially at higher pressures the process of parallel crystallisation is almost entirely supressed in VHDA for all heating rates as compared to literature. To make results directly comparable uHDA was studied under identical conditions as VHDA. One has to note that the protocol of preparation for uHDA varied from that utilised in literature^30^. While in this study uHDA was directly decompressed from 1.6 GPa to the respective pressure at which it was then heated isobarically, uHDA in literature was first decompressed to ambient pressure and then recompressed to higher pressures. During this initial step of decompression microcracks are introduced into the amorphous matrix as has been described previously^35^. This may be the reason for the considerable differences between uHDA studied here and uHDA investigated in literature^30^ regarding crystallisation temperature and especially crystalline phase compositions. The microcracks result in an increase in surface area and may in fact serve as nucleation spots in the amorphous matrix. This may explain lower values of *T*_*x*_ and answer the question why ice IV can be produced almost purely in literature^30^, while in this study there is always a considerable amount of ice XII (or other crystalline phases) next to ice IV.

Here it could be shown that at pressures of *p* > 1 GPa uHDA and VHDA exhibit almost the same crystallisation behaviour. That is, the discrepancy in crystallisation temperature is Δ*T*_*x*_ ≤ 2 K and apparently becomes smaller the higher the pressure. Also, both uHDA and VHDA transform to ice XII almost exclusively no matter the heating rate. This can be reasoned by the circumstance that at pressures *p* > 0.8 GPa HDA will transform to VHDA at elevated temperatures and before crystallising. Thus, at 1.1 and 1.3 GPa the different amorphous phases should be very similar prior to crystallisation. This is also indicated by the fact that the densification step in the volume curves during transformation is of approximately the same size - keeping in mind that both uHDA and VHDA transform almost entirely to ice XII, which will have the same density at a given pressure regardless of the prephase. This observation is supported by relaxation studies with dielectric spectroscopy^36^, indicating that very high-density amorphous ice transforms to an ultraviscous liquid state prior to crystallisation at 1 GPa and *T* > 140 K. Similarly, recent isobaric *in situ* volumetry studies on the relaxation of eHDA at higher pressures have yielded glass transition temperatures *T*_*g*_ of approximately 125 ± 5 K at *p* ≥ 1 GPa^37^.

At lower pressures *p* ≤ 0.8 GPa notable differences in the transformation behaviour can be found. While at low and intermediate heating rates values for *T*_*x*_ are very similar for uHDA and VHDA, they are up to 14 K lower for uHDA when heating with 30 K min^−1^. A possible explanation may be the existence of nano-crystalline domains acting as nucleation seeds within the uHDA matrix which had been proposed to be responsible for the differences in crystallisation behaviour between uHDA and eHDA at lower pressures^31, 32^. It was reasoned that in case of uHDA the crystalline domains only need to grow, whereas in eHDA they first have to form and hence the higher thermal stability against transformation in eHDA of up to 11 K. For VHDA, while at low and intermediate heating rates the time scales may be low enough for crystalline seeds to disappear at 0.7 and 0.8 GPa, this may not be the case when heating with high rates. In this case the nano-crystalline domains may trigger transformation already at much lower temperatures than would be expected.

**Figure 7:**
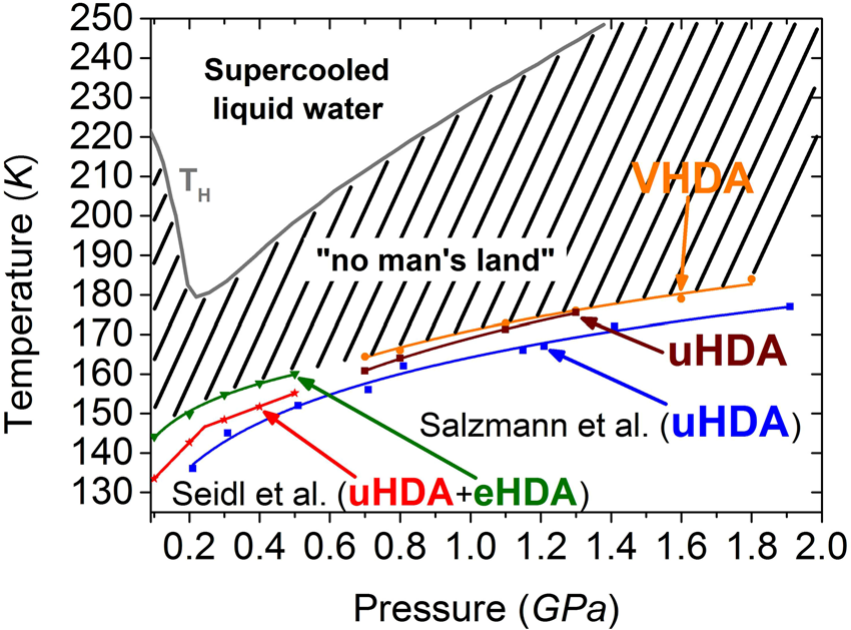
Borders to the no man’s land: *T*_*H*_ from^38^ and *T*_*X*_ for different amorphous ices. In all experiments an intermediate heating rate was applied (2–5 K min^−1^) to determine *T*_*X*_. Studies of Seidl *et al*.^32^ are colour coded in red (eHDA) and green (uHDA). Studies of Salzmann *et al*. in blue (uHDA)^30^. Results of this present study on VHDA in orange and on uHDA in brown.

The size of the no-man’s land and how it was shrinked by the previous work of Seidl *et al*. and the present work is shown in Fig. 7. The crystallisation temperatures of VHDA (orange in Fig. 7) and eHDA (green in Fig. 7) seem to connect continuously as a function of pressure, which indicates that the competing crystallisation channels are similarly suppressed in both eHDA and VHDA. However, there still seems to be room for further improvement, both for eHDA and VHDA, since a few nano-crystalline domains still seem to exist as ice IV formation cannot be avoided entirely, e.g., at 0.7 and 0.8 GPa at low heating rates. Thus, the challenge posed is to eliminate the nano-crystalline domains entirely to shrink no man’s land even further. The present work indicates that the transformation step from uHDA to VHDA governs the slow-down of the slower crystallisation channel. It thus seems sensible to tune the parameters for the uHDA to VHDA transformation in order to aim for complete removal of the competing second crystallisation channel and further narrowing of the no-man’s land. Unfortunately, there is no way of detecting or visualizing these domains directly. The density of nano-crystalline domains can only be inferred indirectly from observations of the rate of the second crystallisation channel, which in the ideal case should become zero once all nano-crystalline domains are removed. In the present work we see a massive slow-down of this second channel, albeit we have not yet reached a rate of zero, i.e., its complete elimination.

## References

[1] Ball P (2008). Water: Water – an enduring mystery. Nature.

[2] Debenedetti, P. G. & Stanley, H. E. Supercooled and Glassy Water. *Physics Today*, 40–46 (2003).

[3] Caupin F (2015). Escaping the no man’s land: Recent experiments on metastable liquid water. Journal of Non-Crystalline Solids.

[4] Petrenko, V. F. & Whitworth, R. W. *Physics of Ice*. (Oxford University Press, 1999).

[5] Zheligovskaya EA, Malenkov GG (2006). Crystalline water ices. Russ. Chem. Rev..

[6] Angell CA (2004). Amorphous Water. Annu. Rev. Phys. Chem..

[7] Loerting T (2011). How many amorphous ices are there?. Phys. Chem. Chem. Phys..

[8] Fuentes-Landete V (2015). Crystalline and amorphous ices. Proceedings of the International School of Physics “Enrico Fermi”.

[9] Salzmann CG, Radaelli PG, Mayer E, Finney JL (2009). Ice XV: A New Thermodynamically Stable Phase of Ice. Phys. Rev. Lett..

[10] Burton EF, Oliver WF (1935). X-ray diffraction patterns of ice. Nature.

[11] Brüggeller P, Mayer E (1980). Complete vitrification in pure liquid water and dilute aqueous solutions. Nature.

[12] Mishima O, Calvert LD, Whalley E (1984). “Melting ice” I at 77 K and 10 kbar: a new method of making amorphous solids. Nature.

[13] Bowron, D. T. *et al*. The local and intermediate range structures of the five amorphous ices at 80 K and ambient pressure: A Faber-Ziman and Bhatia-Thornton analysis. *J. Chem. Phys*. **125**, 194502 (2006). 10.1063/1.2378921 17129118

[14] Loerting T, Salzmann C, Kohl I, Mayer E, Hallbrucker A (2001). A second distinct structural “state” of high-density amorphous ice at 77 K and 1 bar. Phys. Chem. Chem. Phys..

[15] Mishima O, Calvert LD, Whalley E (1985). An apparently first order transition between two amorphous phases of ice induced by pressure. Nature.

[16] Klotz S, Hamel G, Loveday JS, Nelmes RJ, Guthrie M (2003). Recrystallisation of HDA ice under pressure by *in-situ* neutron diffraction to 3.9 GPa. Z. Kristallogr.

[17] Mishima O (1994). Reversible first-order transition between two H2O amorphs at.apprx.0.2 GPa and.apprx.135 K. J. Chem. Phys..

[18] Handle PH, Seidl M, Fuentes-Landete V, Loerting T (2016). *Ex situ* studies of relaxation and crystallization in high-density amorphous ice annealed at 0.1 and 0.2 GPa: Includes response to: “Comment on: ‘Relaxation time of high-density amorphous ice’” by G.P. Johari. Thermochim Acta.

[19] Poole PH, Sciortino F, Essmann U, Stanley HE (1992). Phase Behavior of Supercooled Water. Nature.

[20] Mishima O, Stanley HE (1998). The relationship between liquid, supercooled and glassy water. Nature.

[21] Persico, J. E. Eleventh Month, Eleventh Day, Eleventh Hour: Armistice Day, 1918 World War I and Its Violent Climax. (Random House 2005).

[22] Amann-Winkel K (2016). *Colloquium*: Water’s controversial glass transitions. Reviews of Modern Physics.

[23] Abascal JLF, Vega C (2010). Widom line and the liquid–liquid critical point for the TIP4P/2005 water model. The Journal of Chemical Physics.

[24] Smallenburg F, Filion L, Sciortino F (2014). Erasing no-man’s land by thermodynamically stabilizing the liquid-liquid transition in tetrahedral particles. Nat Phys.

[25] Bergman R, Swenson J (2000). Dynamics of supercooled water in confined geometry. Nature (London).

[26] Beau W, John D (2004). Structural and dynamic studies of water in mesoporous silicas using neutron scattering and nuclear magnetic resonance. Journal of Physics: Condensed Matter.

[27] Manka A (2012). Freezing water in no-man’s land. Phys. Chem. Chem. Phys..

[28] Sellberg JA (2014). Ultrafast X-ray probing of water structure below the homogeneous ice nucleation temperature. Nature.

[29] Laksmono H (2015). Anomalous Behavior of the Homogeneous Ice Nucleation Rate in “No-Man’s Land”. J. Phys. Chem. Lett..

[30] Salzmann CG, Mayer E, Hallbrucker A (2004). Effect of heating rate and pressure on the crystallization kinetics of high-density amorphous ice on isobaric heating between 0.2 and 1.9 GPa. Phys. Chem. Chem. Phys..

[31] Seidl M, Amann-Winkel K, Handle PH, Zifferer G, Loerting T (2013). From parallel to single crystallization kinetics in high-density amorphous ice. Phys. Rev. B.

[32] Seidl M, Fayter A, Stern JN, Zifferer G, Loerting T (2015). Shrinking water’s no man’s land by lifting its low-temperature boundary. Phys. Rev. B.

[33] Seidl, M. *et al*. High-performance dilatometry under extreme conditions. *Proceedings of the 6th Zwick Academia Day 2015* (2015).

[34] Loerting T (2006). Amorphous Ice: Stepwise Formation of Very-High-Density Amorphous Ice from Low-Density Amorphous Ice at 125 K. Phys. Rev. Lett..

[35] Salzmann CG (2006). Isobaric annealing of high-density amorphous ice between 0.3 and 1.9 GPa: *in situ* density values and structural changes. Phys. Chem. Chem. Phys..

[36] Andersson O (2005). Relaxation Time of Water’s High Density Amorphous Ice Phase. Phys. Rev. Lett..

[37] Handle PH, Loerting T (2016). Dynamics anomaly in high-density amorphous ice between 0.7 and 1.1 GPa. Phys. Rev. B.

[38] Mishima O, Stanley HE (1998). Decompression-induced melting of ice IV and the liquid-liquid transition in water. Nature.

[39] Engelhardt H, Kamb B (1981). Structure of ice IV, a metastable high-pressure phase. J. Chem. Phys..

[40] Kamb B, Prakash A, Knobler C (1967). Structure of ice V. Acta Cryst.

[41] Kamb B (1965). Structure of ice VI. Science.

[42] Koza M, Schober H, Tölle A, Fujara F, Hansen T (1999). Formation of ice XII at different conditions. Nature.

[43] Amann-Winkel, K. *et al*. Water's controversial glass transitions. *Rev. Mod. Phys.* **88**, 011002 (2016).

